# Exfoliative Cheilitis as a Manifestation of Factitial Cheilitis

**DOI:** 10.7759/cureus.2565

**Published:** 2018-05-02

**Authors:** Raghavendra L Girijala, Rachel Falkner, Scott R Dalton, Brent D Martin

**Affiliations:** 1 College of Medicine, Texas A&M; 2 Department of Pathology and Dermatology, Brooke Army Medical Center, Fort Sam Houston, USA; 3 Department of Oral and Maxillofacial Pathology, San Antonio Military Medical Center

**Keywords:** anxiety, cheilitis, depression, exfoliative, factitial, psychiatric

## Abstract

Factitial cheilitis is a rare diagnosis of exclusion that occurs most frequently in young women with a history of anxiety disorders and recent psychosocial stressors. It presents as continuous keratinaceous build-up, crusting, and desquamation of the lips, consistent with exfoliative cheilitis. Affected areas can progress to superinfection with Staphylococcus aureus or Candida albicans. We report a case of a 23-year-old woman who presented with diffuse hyperkeratosis of the upper and lower lips that was initially suspected to be allergic or irritant contact dermatitis based on clinical examination. Clinical and histologic correlation of two separate biopsies plus a negative infectious workup led to the consideration of a factitial etiology. Through open and direct communication between the patient and the provider, the appropriate diagnosis was discerned. Referral for the psychiatric symptoms as well as management of the same resulted in complete resolution of her lip findings. This case highlights the importance of considering factitial cheilitis as the etiology of exfoliative cheilitis, especially in the presence of concomitant psychiatric disorders.

## Introduction

Factitial or factitious cheilitis is a diagnosis of exclusion that often presents in young women with a history of psychiatric illness [1]. The differential diagnosis in patients presenting with exfoliative and inflammatory changes of the lip(s) is broad, including, but not limited to, immune-mediated, inflammatory, infectious, and neoplastic etiologies [2-3]. In patients with non-specific clinical and histopathological findings recalcitrant to pharmacologic therapy and standard of care management, factitial cheilitis should be considered. We report a 23-year-old woman who presented with exfoliative cheilitis that was eventually diagnosed with factitial cheilitis.

## Case presentation

**Figure 1:**
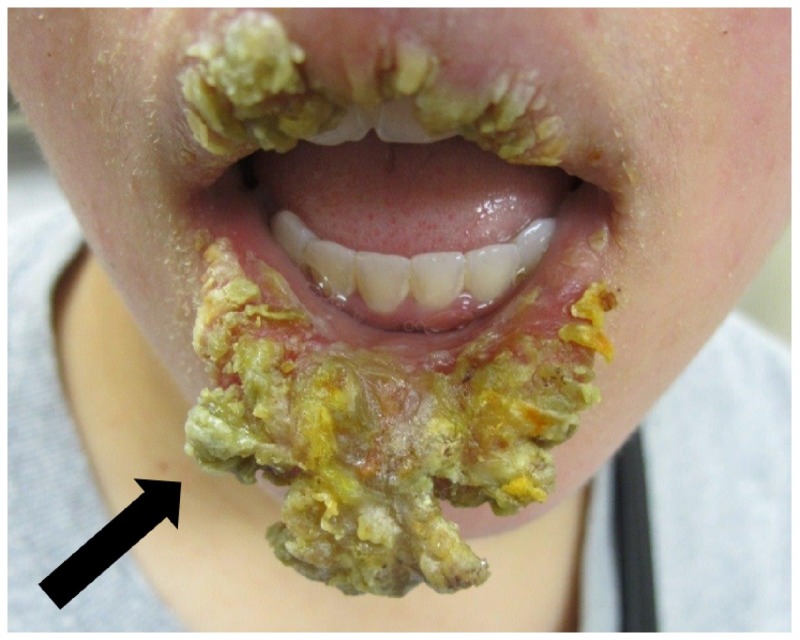
Physical examination findings consistent with severe exfoliative cheilitis. Thick yellow crust on the bilateral upper and lower lips with edematous change, erosions, and tenderness to palpation. The mucosal lip and vermillion border are spared.

A 23-year-old woman with a history of adjustment disorder and previously treated anxiety disorder, not otherwise specified, presented to the dermatology clinic with a two-month history of painful, cracked, and peeling lips that had been unresponsive to oral acyclovir or valacyclovir. Examination revealed thick, yellow keratinaceous crusting on the upper and lower lips, sparing the mucosal lip and vermilion border, with associated edema, erosion, and tenderness to palpation (Figure 1). Notably, minimal contact with the keratinaceous material and crusted plaque on physical examination resulted in complete sloughing and revealed a moist base and a nearly normal lip underneath.

Consequently, the histopathological evaluation of a 4 mm punch biopsy from the debrided left lower lip was nonspecific, showing only features of chronic lymphocytic mucositis with spongiosis. Staining for fungal organisms using the Grocott's methenamine silver and periodic acid–Schiff methods was negative, and Treponemal/Fite stains were negative for bacterial organisms. In-situ hybridization for human papillomavirus testing (subtypes 6/11, 16/18, 31/33) was also negative. A wound culture from the lower lip was positive for methicillin-sensitive Staphylococcus aureus.

While the biopsy findings were not specific, the histopathologic pattern of spongiotic mucositis, in conjunction with the clinical features, suggested an exuberant irritant contact dermatitis—in this case, further investigation revealed repeated self-injurious behaviors to the lips, supporting a diagnosis of factitial cheilitis. The patient was treated for secondary impetiginization with anti-staphylococcal antibiotics and topical antibiotic ointment for two weeks, counseled on avoidance of licking her lips, and referred back to her behavioral health specialist for management of her unspecified anxiety disorder. Notably, she had previously been treated for anxiety, but was lost to follow-up prior to presentation at our clinic. However, she noted that recent life stressors had caused exacerbation of her anxiety symptoms, which preceded the onset of her lip findings. The patient reported complete resolution of her lip symptoms within one month of psychiatric treatment and cessation of lip licking.

## Discussion

Factitial cheilitis is a rare condition characterized by cyclic and continuous peeling of excess keratin due to behaviors such as persistent lip licking, sucking, biting, and picking [2]. In addition to the excess keratin, patients present with dry and scaly lips that can have associated crusting, fissuring, and hemorrhage due to the cyclical nature of the self-harming behaviors [2, 4]. Pain and difficulty smiling, speaking, or eating can occur in severe cases [5]. Other significant problems include aesthetic concerns and self-esteem-related issues, all which contribute to the ongoing factitial behavior. As with our patient, factitial cheilitis can present as exfoliative cheilitis; it is important to note that the former describes the underlying etiology, whereas the latter describes the disease process [2]. Consequently, exfoliative cheilitis may simply represent the histologic diagnosis, and only through correlation with clinical and psychosocial behaviors can a definitive diagnosis of factitial cheilitis be rendered. 

The distinction between exfoliative and factitial cheilitis is imperative to understand; if factitial etiologies are not considered in the differential diagnosis, the contributing behaviors can be missed for many years. In the interim, patients can undergo multiple trials of empirical therapy and repeated nonspecific biopsies, all while incurring significant medical costs. Understandably, patients grow increasingly frustrated with their cosmetically disfiguring lip lesion, while the medical community remains perplexed [6]. Indeed, factitial cheilitis has frequently been initially diagnosed as exfoliative cheilitis; further investigation has often demonstrated that many cases of exfoliative cheilitis were, in fact, factitial in nature [3, 4, 7].

Affected patients tend to have underlying psychiatric illnesses that are either undiagnosed or not appropriately managed; this includes anxiety, depression, attention-seeking behavior, obsessive-compulsive tendencies, delusions, hallucinations related to the skin, dissociative disorder, personality disorder, and a history of child abuse [1, 6]. Furthermore, factitial habits tend to affect adolescent and young adult women; triggering factors such as a recent psychosocial stressor have been temporally linked to the onset of self-injurious behaviors predisposing one to factitial cheilitis [1, 6]. In contrast, exfoliative cheilitis does not demonstrate gender predilection [5].

Peeling of the lips tends to affect the lower lip more than the upper lip if both are affected; the vermillion zone of the lips is also involved [2]. Although the symptoms, such as pain, dryness, itching, burning, and bleeding, tend to be continuous, lesions in factitial cheilitis often present at different stages. The clinical presentation depends on the intensity and frequency of the habit, as well as the waxing and waning nature of the factitial injury. [2]. Excess build-up of keratin and crusting eventually results in desquamation, which can either be a natural byproduct of the habit or manually induced by picking or peeling [2]. Notably, and somewhat surprisingly, despite the alarming clinical presentation, the underlying lips are often cosmetically normal, indicating a benign, likely reactive, etiology. Cases involving a hemorrhagic or peculiar pattern of crusting should be evaluated for malignancy [1]. Finally, given the predisposition to fissured lips, superinfection with Staphylococcus aureus or Candida albicans needs to be considered [3].

Patients presenting with hyperkeratotic or crusted lip lesions must undergo a thorough history and physical examination due to the broad differential diagnosis. In no particular order, conditions that should be considered include actinic cheilitis, allergic or irritant contact dermatitis, cheilitis glandularis, isolated lichenoid mucositis, malignancy, photosensitivity reactions, vitamin A toxicity, and fungal and bacterial etiologies [1, 3, 8].  

Isolated irritant or allergic contact dermatitis of the lips most commonly involves the vermilion border rather than oral mucosa and can present with dryness, edema, and fissuring of the lips [9]. The vast majority of patients are females who wear lipstick; however, this can also occur in patients with exposure to sunscreen lip balms, dentifrices or other dental preparations, and topical medications. Removal of the irritant or allergenic substance is key to the resolution of their symptoms [9].

Actinic cheilitis, which largely affects an older age group than factitial cheilitis, is associated with a history of chronic sun exposure [2]. Physical examination demonstrates crusting, but also dry, scaly regions that develop into white-gray plaques with a risk of malignant degeneration. However, clinical findings can also include thin, fragile skin, a stark contrast from findings in factitial cheilitis [3].

Cheilitis glandularis displays more diffuse swelling due to enlarged salivary glands; in addition, removal of serum crusting demonstrates salivary gland openings on the lips with a pebble-like feeling on palpation [3]. On histopathology, cheilitis glandularis demonstrates a mixed inflammatory pattern with plasma cells, histiocytes, and lymphocytes that surround and invade the glands [10]. Finally, while Candida cheilitis can be associated with hemorrhage and crusting like factitial cheilitis, hyperkeratotic scale is not a feature [2].

When attempting to discern the diagnosis, workup in patients presenting with crusting lip lesions should include bacterial and fungal cultures to rule out primary infectious etiologies or superinfection [1]. Furthermore, histopathological evaluation should be conducted to rule out malignancy [1]. In cases where factitial cheilitis is not initially suspected, the exclusionary benefits of failed therapies can be beneficial and contribute to the eventual diagnosis. Therapies such as antifungals and antibiotics will be ineffective beyond eradication of superinfection, while vitamin supplementation only assists in ruling out hypervitaminosis A [2]. Topical, intralesional, and systemic steroid treatments have not demonstrated significant effects. Similarly, cryotherapy and radiation therapies have limited efficacy [2].

To reliably recognize and diagnose factitial cheilitis, open and honest communication between the clinician and the patient, as well as close coordination between the clinician and the pathologist is required. In our case, the associated histology was rather unremarkable, with only nonspecific features identified. Conversely, the clinical presentation, as evidenced by the clinical photo, is quite remarkable and worrisome. The correlation of clinical and histopathologic findings, especially if they appear discordant, may be the primary driver behind further investigation. If the patient denies factitial behavior, the diagnosis of factitial cheilitis can be even more challenging, as providers remain perplexed by the diffuse, cyclic, and refractory nature of the process. With these patients, multiple biopsies of the lips may occur, yielding the same underwhelming nonspecific histopathologic features. Indeed, it is important to note that factitial cheilitis cannot be made based on histopathologic features alone.

Ultimately, the management of factitial cheilitis is predicated on a multi-disciplinary approach involving psychiatry, dermatology, oral medicine/pathology, and primary care physicians, which allows for accurate, timely diagnosis and coordination of psychotherapy sessions and pharmacotherapy [6]. Given the multitude of psychiatric conditions associated with factitial cheilitis, the specific drug and psychotherapy regimens used will depend on the individual patient. Notably, patients with underlying mood or anxiety disorders tend to respond more than those with personality disorders due to the latter’s recalcitrance to medical treatment [1].

## Conclusions

Factitial cheilitis is a chronic condition characterized by crusting, hyperkeratosis, and ulceration afflicting patients with self-injurious behaviors such as lip biting. While it is of benign origin, it can be painful or cosmetically disfiguring for many patients. In patients presenting with exfoliative cheilitis, a thorough history should be taken for concomitant psychiatric illness to determine if factious etiologies are involved.
